# Aluminum-ceramic composites for thermal management in energy-conversion systems

**DOI:** 10.1038/s41598-018-36270-x

**Published:** 2018-12-14

**Authors:** Jehong Park, Seungchan Cho, Hansang Kwon

**Affiliations:** 1 Next-Generation Materials Co., Ltd. (NGM), 1401, Centum Science Park, 79 Centum jungang-ro, Haeundae-gu, Busan 48058 Republic of Korea; 2 Korea Institute of Materials Science, 797 Changwondaero, Changwon, Gyeongnam 51508 Republic of Korea; 3 Department of Materials System Engineering, Pukyong National University, Building-7, 365 Sinseon-ro, Busan, 48547 Republic of Korea

## Abstract

The most important property of energy-conversion ceramics in high-power lighting devices based on laser diodes (LDs) is thermal durability because high-energy LDs act as excitation and heat sources for ceramics. Herein, aluminum-ceramic composites (ACCs) are introduced for the manipulation of heat generated during high-power lighting. The cerium-doped aluminum garnet (YAG:Ce) phosphor is selected as the energy-conversion ceramic material. The ACCs have an all-in-one structure bridged by a low-melting glass material. In ACCs, the heat flow from the ceramic to Al is manipulated by a heat-flux throttling layer (TL) comprised of Al and glass. During high-power lighting operation, the input-output temperature differences (T_in_ − T_out_) between the ceramic layer (input heat) and end face of the Al layer (output heat) are 13 and 3.9 °C in the absence and presence of the TL, respectively. A lower T_in_ − T_out_ means less heat is loss during heat flow from the ceramic to the metal due to the temperature gradient created by inserting the TL. The results provide a potential application for multi-energy-conversion systems, i.e., optical to heat and heat to electric energy, in terms of heat flow manipulation.

## Introduction

Thermal management is an essential technology in high-power electronic devices^1–7^. Waste heat is available as an energy source, and overheating of functional materials reduces material performance and causes device failure. High-power laser diode (LD)-based lighting devices are an emerging technology for next-generation lighting because their high-brightness and compactness compared to those of light-emitting diode (LED)-based lighting devices^8–14^. In the simplest architecture of LD- or LED- based lighting, energy-conversion ceramics are combined with blue-emitting devices such as an LD and LED^15–17^. The ceramic converts part of the blue light into yellow light, and the resulting mixture of blue and yellow light creates white light. The thermal durability of the ceramic is a prerequisite for stable lighting in LD-based high-power lighting because the ceramic is exposed to thermal energy from the high-energy LD^9,13,14^. In general, the performance of an energy-conversion ceramic degrades above a temperature threshold due to phonon-electron interactions^18^. Therefore, effective thermal management of the ceramic is required for normal functioning of lighting devices. However, the ceramic is limited by thermal conductivity for effective heat dissipation^9,13,14,19–21^. Recently, we reported thermally robust metal-ceramic composites as an approach to overcome the limitations of ceramics^22^. Interestingly, the results showed that the heat generated from the ceramic can be used as an energy source during lighting operations by coupling the metal-ceramic composites with a thermoelectric module. In energy-management systems, heat is an important energy-conversion source. For example, electric vehicles (EVs) have been recently developed to meet environmental regulations as an alternative for traditional vehicles^23–27^. The heating system in EVs is important because EVs do not have a heat sources, such as the engines used in conventional gasoline vehicles. Therefore, EVs require heating units and use an electric heater to convert electric energy into heat through conductive heating materials. However, the electric heater in EVs has limitations and disadvantages in terms of its energy consumption. In this respect, managing the small amount of waste heat from the operation of electronic devices in EVs is important. Lighting devices are essential components in vehicles and industry. The waste heat generated from the operation of lighting systems can be used in energy-management systems^27–29^. Our previous results show the feasibility of using heat from high-power lighting as an energy source^22^.

Herein, aluminum-ceramic composites (ACCs) are introduced to manipulate heat generated during high-power lighting. The cerium-doped aluminum garnet (YAG:Ce) phosphor was selected as the energy-conversion ceramic material. The ACCs have an all-in-one structure bridged by a low-melting glass material. In ACCs, the heat flow from the ceramic to Al is manipulated by a heat-flux throttling layer (TL) comprised of Al and glass. To investigate the temperature profile of the ACCs, the ceramic is excited with a high-power blue LD (455 nm, 4 W), and the temperature distribution in the ACCs is monitored.

## Results and Discussion

**Figure 1:**
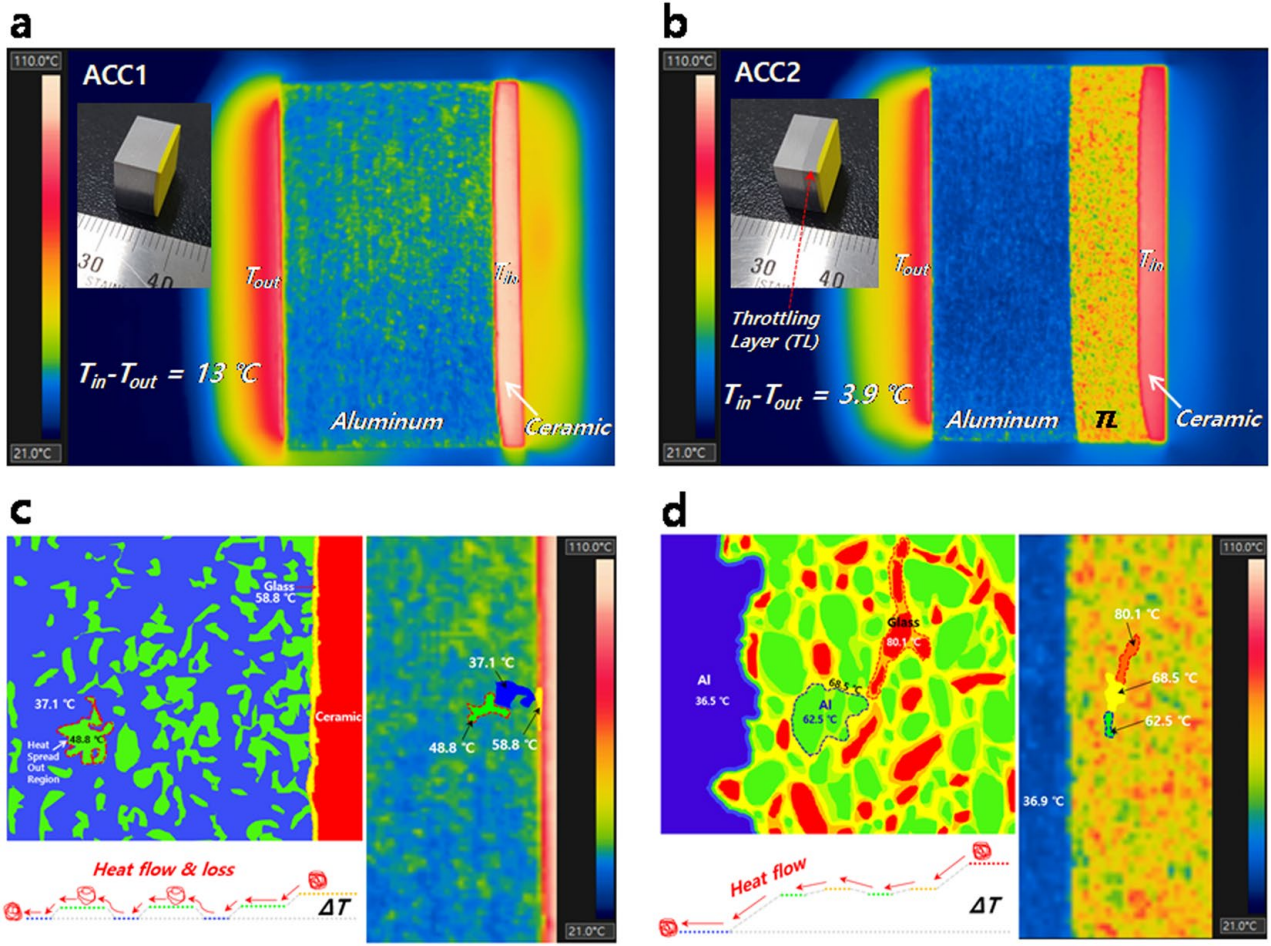
Temperature distribution of ACCs under a 4 W-blue LD. (**a**) Temperature distribution and photo image of ACC1. (**b**) Temperature distribution and photo image of ACC2. The ceramic layer comprises 50 vol% YAG:Ce and 50 vol% glass. The Al layer is directly fused with the ceramic layer in ACC1. ACC2 has a TL between the Al and ceramic layers, and the TL comprises 50 vol% Al and 50 vol% glass. (**c**) Schematic diagram for the temperature distribution and heat flow (left) in the Al region of ACC1 and a magnitude image (right) of the temperature distribution in the Al region. The temperature of the glass region at the interface between the Al and ceramic region is 58.8 °C. The temperatures of the Al regions are 48.8 (HSOR) and 37.1 °C, respectively. (**d**) Schematic diagram of the temperature distribution and heat flow (left) in the TL region of ACC2 and a magnitude image (right) of the temperature distribution in the TL region. The temperatures of the glass and Al regions are 80.1 (hot zone) and 62.5 °C, respectively, in the TL. The temperature of the glass region adjacent to the Al is 68.5 °C.

Figure 1a,b present the temperature distribution of ACCs that consist of aluminum (Al) and YAG:Ce phosphors as a high thermal conductivity metal and energy-conversion ceramic, respectively, under a 4 W LD that hits the ceramic face. The LD can be simultaneously used as an excitation and heat source for the ceramic. The setup for monitoring the temperature of each area in the ACCs is presented in Supplementary Fig. S1, and the high bright-white lighting is generated by the combination of the ceramic and LD during monitoring. The inset images of Fig. 1a,b show ACCs fabricated by the spark plasma sintering (SPS^30^) process, as presented in Supplementary Fig. S2. During the SPS process, the glass has a fluidity, as seen in the shrinkage displacement of Supplementary Fig. S2. Consequently, the glass wraps around the ceramic particles, and the interface between the Al and the ceramic is fused by the glass, as seen in the scanning electron microscope (SEM) and element mapping images in Supplementary Fig. S3. The Al and ceramic are completely fused, creating an all-in-one structure bridged by a low-melting glass. The interesting feature of ACC2 is the positioning of the TL between the Al and ceramic layers, as presented in Fig. 1b and Supplementary Fig. S4. The TL is comprised of Al and glass. In ACC2, 50 vol% of Al is in the TL, and glass wraps around the Al particles, as presented in the SEM images in Supplementary Fig. S4.

**Figure 2:**
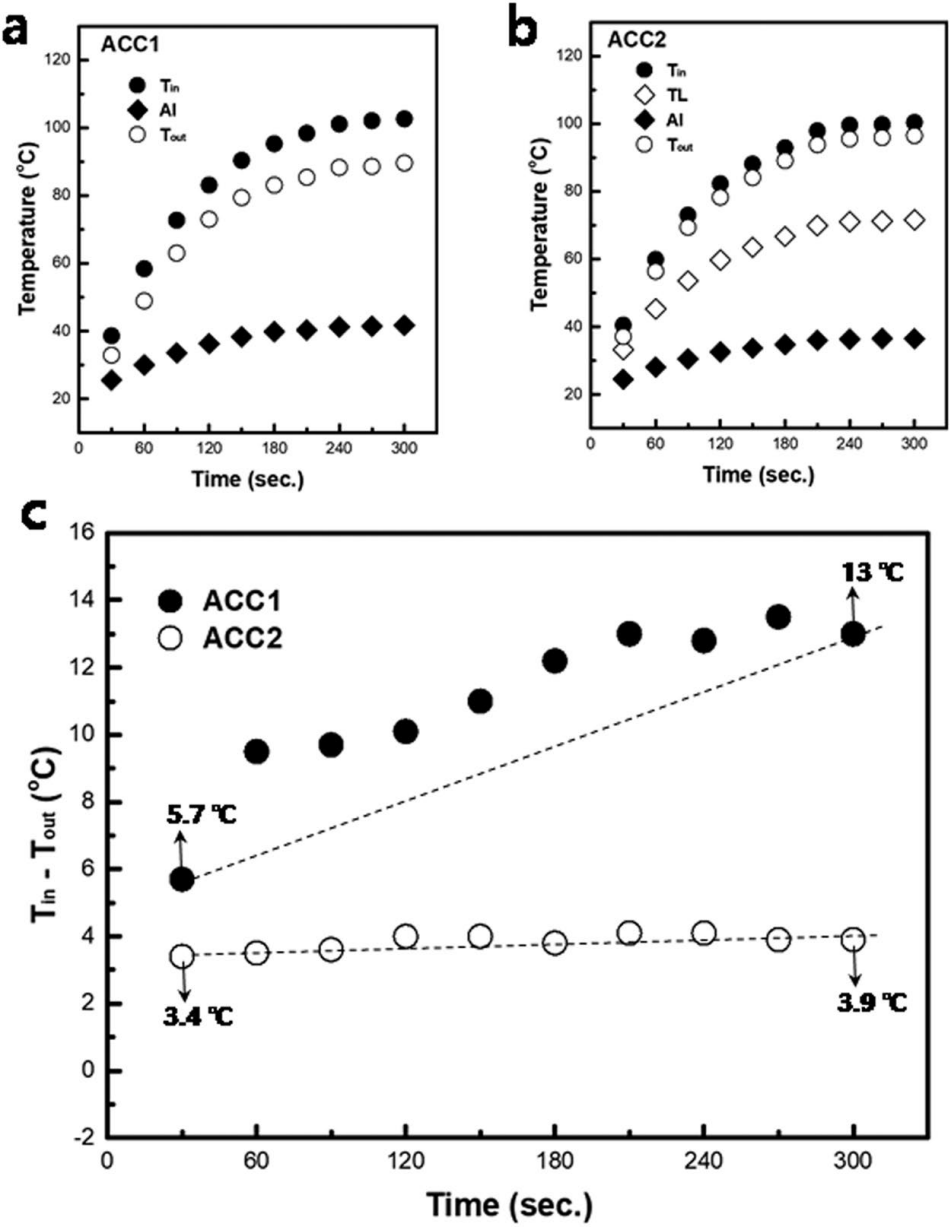
Temperature profiles of the ACCs under a 4 W-blue LD over time (The data were collected from images of the temperature distribution, as presented in Supplementary Figs S5 and S6. The temperature values of the each region in the ACCs were averaged in the area of the each region as seen in Supplementary Fig. S1.). The T_in_ and T_out_ are the temperatures of the ceramic and the end of the Al region, respectively (see Fig. 1). (**a**) Temperature profiles of each region in ACC1. (**b**) Temperature profiles of each region in ACC2. (**c**) Temperature input-output difference (T_in_ − T_out_) of ACC1 and ACC2.

Inserting the TL between the Al and ceramic layers results in different, temperature distributions for ACC1 and ACC2. The input-output temperature differences (T_in_ − T_out_) of ACC1 and ACC2 are 13 and 3.9 °C, respectively, which means that heat loss probably occurs in ACC1 as the heat flows from the ceramic to the Al. The heat loss mainly occurs in the Al region of ACC1 because the Al region of ACC1 does not have a temperature gradient to accelerate the heat flow. The heat flux in the Al region of ACC1 has a drift state, and a certain amount of the heat flux can diffuse into the air through the heat spread out region (HSOR), as presented in Fig. 1c. Heat dissipation from the ceramic is obviously important, but the delivery of the generated heat is also important to utilize the heat as an energy source. The TL in ACC2 can create a temperature gradient that has an important role in heat flowing to the Al region. The red and green areas in the TL region are glass and Al, respectively, as presented in Fig. 1b,d (see Supplementary Fig. S4). The glass areas create a hot zone, and the Al wrapped by glass does not have a sufficient network for heat dissipation; nevertheless, the heat accumulated in the glass can be gradually transferred to the cold Al areas. In Fig. 1b,d the yellow color region of the TL represents a heat-transfer pathway that steadily dissipates the generated heat from the ceramic. Ultimately, the TL provides a temperature gradient to accelerate the heat flow in ACC2, and the accelerated heat flux arrives at the end of the Al, minimizing the heat flux loss in the Al region. The temperature profiles of ACC1 and ACC2 under 4-W LD over time are presented in Fig. 2a,b (see Supplementary Figs S5 and S6). The T_in_ − T_out_ of ACC1 increases and becomes unstable over time, whereas the T_in_ − T_out_ of ACC2 is stable, as presented in Fig. 2c. This result shows that the TL can affect the heat flow toward the Al region. The T_in_ − T_out_ of ACC2 is stable with minimal change because ACC2 always has a gradient to accelerate the heat flux in the area between the TL and Al region. Thus, heat generated from the ceramic is transferred to the end of the Al area through the TL, minimizing the heat loss. In the case of ACC1, the heat flux in the Al region increases over time because the Al region in ACC1 does not have a temperature gradient to accelerate the heat flux. Thus, the heat flux drifts, and a certain amount of the heat flux diffuses into the air through the HSOR in the Al region, as seen in Fig. 1c.

**Figure 3:**
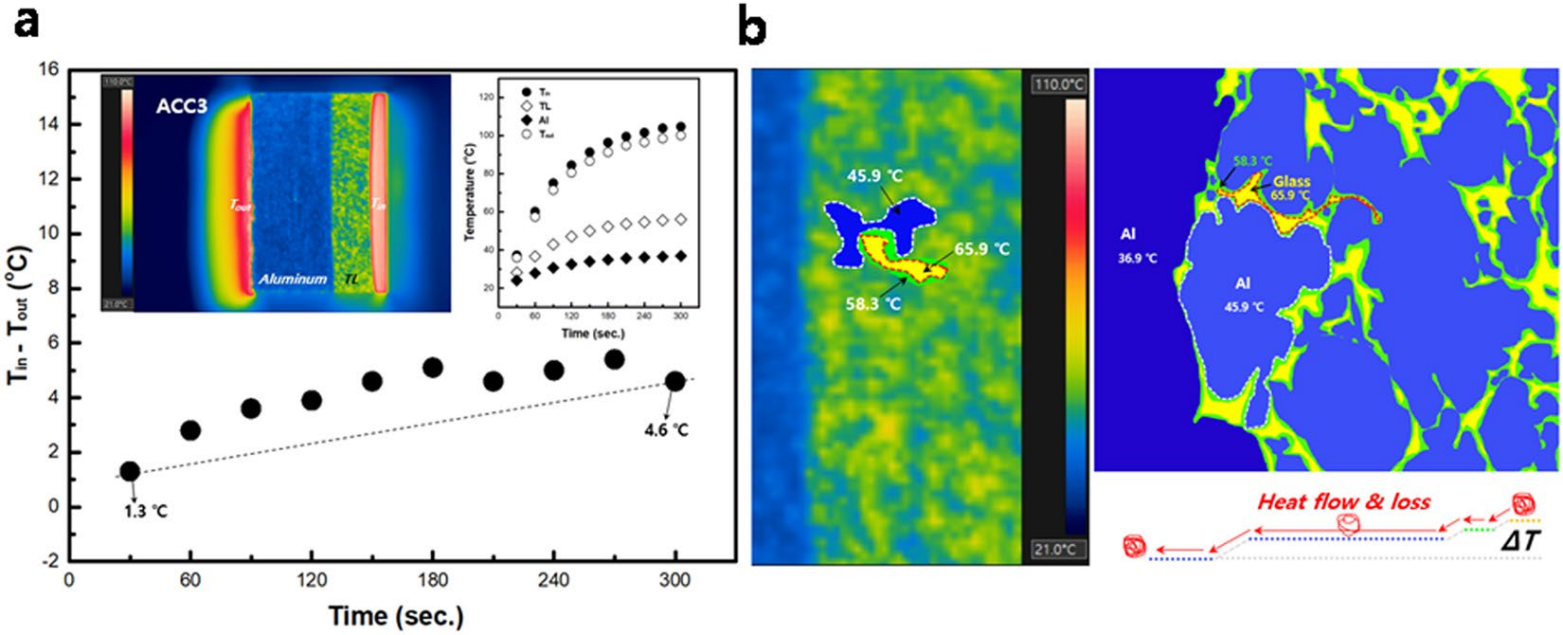
Temperature distribution of ACC3. The TL comprises 75 vol% Al and 25 vol% glass. (**a**) Temperature input-output difference (T_in_ − T_out_) of ACC3. Insets are the temperature distribution (left) and profile for each region (right, data collected from images of the temperature distribution, as presented in Supplementary Fig. S8.). (**b**) Magnitude image (left) and schematic diagram of the temperature distribution and heat flow (right) in the TL region of ACC2. The temperatures of the glass and Al regions are 65.9 and 45.9 °C, respectively, in the TL. The temperature of the glass region adjacent to the Al is 58.3 °C.

Figure 3a presents T_in_ − T_out_ for ACC3 with an Al-network TL (75 vol% Al, see Supplementary Fig. S7), and the inset of Fig. 3a shows the temperature distribution (left side) and temperature profile over time (right side, see Supplementary Fig. S8) for each region. The results show that the T_in_ − T_out_ of ACC3 increases over time and is slightly higher than that of ACC2 at 300 sec. For the TL in ACC3, as presented in Fig. 3b, the Al can form a network for heat dissipation, and the heat flow mainly occurs via the Al network, in contrast to ACC2. During the heat flow, the heat flux can slightly drift in the Al particle network in the TL of ACC3, causing a small heat loss similar to that observed with ACC1. The temperature gradient between the TL and Al region of ACC3 (ΔT = 19.2 °C) is smaller than that of ACC2 (ΔT = 35.1 °C), but the heat generated from the ceramic can be more easily transferred through the TL compared to that in ACC1, which does not have a temperature gradient.

**Figure 4:**
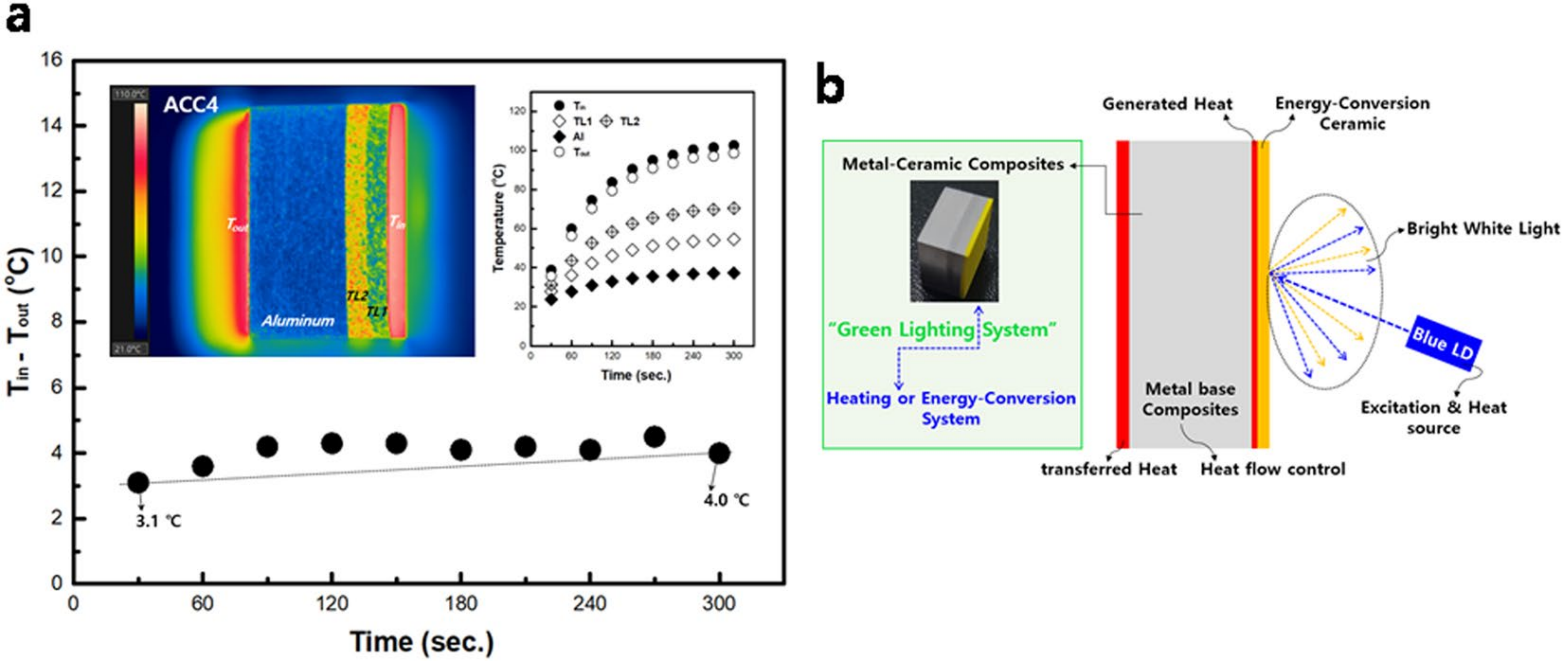
(**a**) Temperature input-output difference (T_in_ − T_out_) of ACC4. Insets are the temperature distribution (left) and profile for each region (right, data collected from images of the temperature distribution, as presented in Supplementary Fig. S10). TL1 comprises 75 vol% Al and 25 vol% glass. TL2 comprises 50 vol% Al and 50 vol% glass. (**b**) Schematic diagram of the “green lighting system” using metal-ceramic composites for applications such as EV, airplanes and indoor and, outdoor lighting. Metal materials can be selected based on the application, and the heat flow can be controlled by the design of the control layers.

Figure 4a presents the results of ACC4 with two TLs. The first and second TL have 75 vol% Al (TL1) and 50 vol% Al (TL2), respectively, as presented in Supplementary Fig. S9. TL2 merged with the Al region to create a high temperature gradient, as seen with ACC2. The T_in_ − T_out_ of ACC4 is similar to that of ACC2, which has a stable T_in_ − T_out_ compared to that of ACC3. The results reveal that the temperature gradient is important for manipulating the heat flow and that the temperature gradient can be easily controlled by the material design.

## Conclusion

In summary, the heat flow from the ceramic to the metal can be manipulated by the heat-flux TL in ACCs. The TL has an important role in transferring the heat flux to the Al region, and the heat flux is easily transferred by the TL, which builds sufficient temperature gradient in ACCs. These results imply that the heat flow in ACCs is manipulated by controlling the composition and position of the TL. In terms of heat flow manipulation, our concept is unlike other heat spreading mechanisms that require heat dissipation from the heat source. We expect that manipulating the heat from lighting can be applied to heating and energy-conversion systems, as presented in Fig. 4b. By modifying the material design for different applications, this “green lighting system” could be applied to EV and airplanes as well as indoor and outdoor lighting with energy recycling.

## Methods

### Materials

The following powders were used as starting materials for the experiments: pure Al (ECKA Granules, Germany, particle size less than 100 µm, density of 2.70 g/cm^3^), borosilicate glass (B_2_O_3_-SiO_2_-Al_2_O_3_, BASS Co., Ltd., Korea, particle size less than 10 µm, glass-transition point of 470 °C, softening point of 532 °C, density of 2.40 g/cm^3^), and YAG:Ce phosphor (L-STONE Co., Ltd., Korea, particle size less than 15 µm, density of 4.56 g/cm^3^). The powder mixtures (YAG:Ce/glass = 0.50/0.50, Al/glass = 0.50/0.50 and Al/glass = 0.75/0.25) were prepared by mixing the powders in an agate mortar. All powder mixtures were mixed by volume fraction.

### Fabrication of the Aluminum-Ceramic Composites (ACCs)

The prepared powders were stacked in a cylindrical graphite die with a diameter of 15 mm in the order pure Al (metal layer), mixture of Al/glass (throttling layer; TL) and YAG:Ce/glass (ceramic layer). The stacked powders were sintered using a customized SPS machine, model SPS-321 Lx (Fuji Electronic Industrials Co., Ltd., Japan). SPS was conducted at 550 °C (heating rate of 100 °C/min.) for 1 min at a pressure of 30 MPa. The fabricated ACCs were round disks with a diameter of 15 mm and a thickness of 7.0 mm.

### Morphology

The morphology of the fabricated ACCs was observed using a Tescan Vega scanning electron microscope (SEM, Czech Republic) equipped with a Horiba Emax energy-dispersive spectrometer (EDS, Japan).

### Temperature Distribution

The temperature distribution of the ACCs was observed using a high-resolution infrared camera (FLIR T420, Sweden) under a high-power blue LD (4 W, 445 nm, spot size of 24 mm^2^, LASEVER LSR445CP, China), as seen in Supplementary Fig. S1. SPS samples were treated in an area of 10 mm × 10 mm to observe the temperature distribution in each region. The temperature distribution of all samples was observed at fixed position under top view during the operation of the LD.

## Electronic supplementary material

Supplementary_Information
