# Supplementary material for: Aluminum-ceramic composites for thermal management in energy-conversion systems
Source: Sci Rep. 2018 Dec 14;8:17852. doi: 10.1038/s41598-018-36270-x (PMC6294771; doi:10.1038/s41598-018-36270-x)
Supplement: Supplementary file 1 — Supplementary_Information [file 41598_2018_36270_MOESM1_ESM.doc]

Supplementary Information

**Aluminum-ceramic composites for thermal management in energy-conversion systems**

Jehong Park ^1^, Seungchan Cho ^2^ and Hansang Kwon ^1,3,*^

^1^ Next-Generation Materials Co., Ltd. (NGM), 1401, Centum Science Park, 79 Centum jungang-ro, Haeundae-gu, Busan, 48058, Republic of Korea

^2^ Korea Institute of Materials Science, 797 Changwondaero, Changwon, Gyeongnam, 51508, Republic of Korea

^3^ Department of Materials System Engineering, Pukyong National University, Building-7,

365 Sinseon-ro, Busan, 48547, Republic of Korea

*Correspondence and requests for materials should be addressed to

H. K (email:[kwon13@pknu.ac.kr](mailto:kwon13@pknu.ac.kr))

This file includes several supplementary images (Figure S1 to S10) to provide additional information for the manuscript.


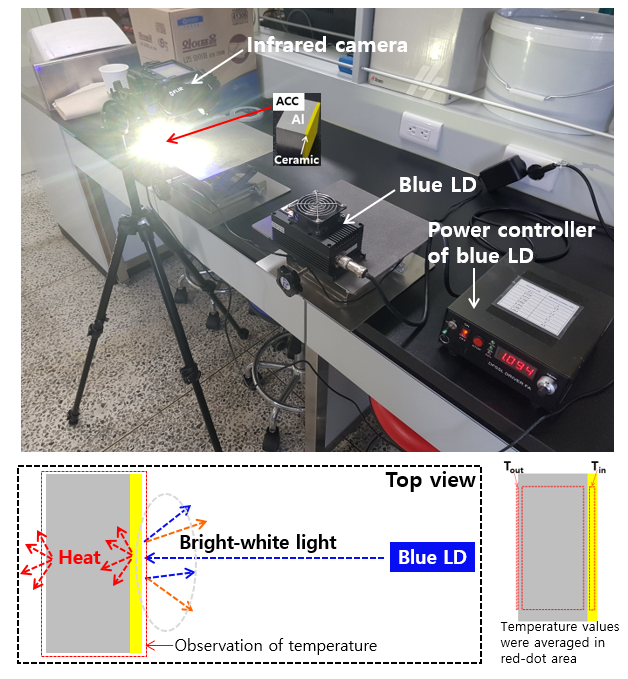


**Figure S1.** Experimental setup for monitoring the temperature distribution of ACCs during the operation of a 4 W-blue LD. The temperature distribution of ACCs was monitored by infrared camera and the temperature of ACCs was observed at fixed position under top view during the operation of the LD. The temperature values of the each region in the ACCs were averaged in the area of the each region.


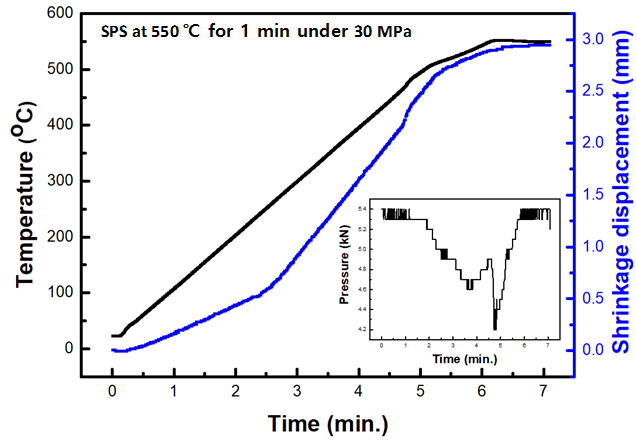


**Figure S2.** Temperature, displacement and pressure (inset) profiles during SPS fabrication of ACC1.

Note: Up to 400 ℃, the sample slowly shrinks due to powder rearrangement, and then, the sample suddenly shrinks (a pressure reduction was observed at the same time, as seen in the inset) in the range from 400 to 500 ℃ due to glass softening.


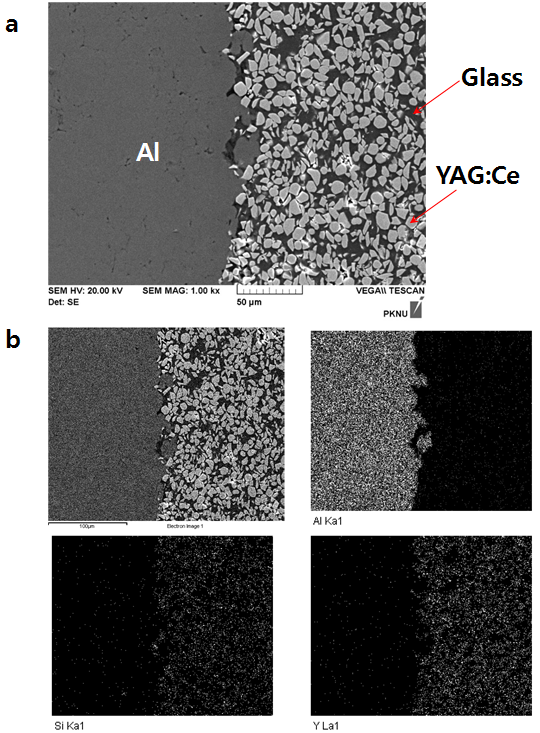


**Figure S3.** (a) SEM and (b) Element mapping images of ACC1. The ceramic layer comprises 50 vol% YAG:Ce and 50 vol% glass.


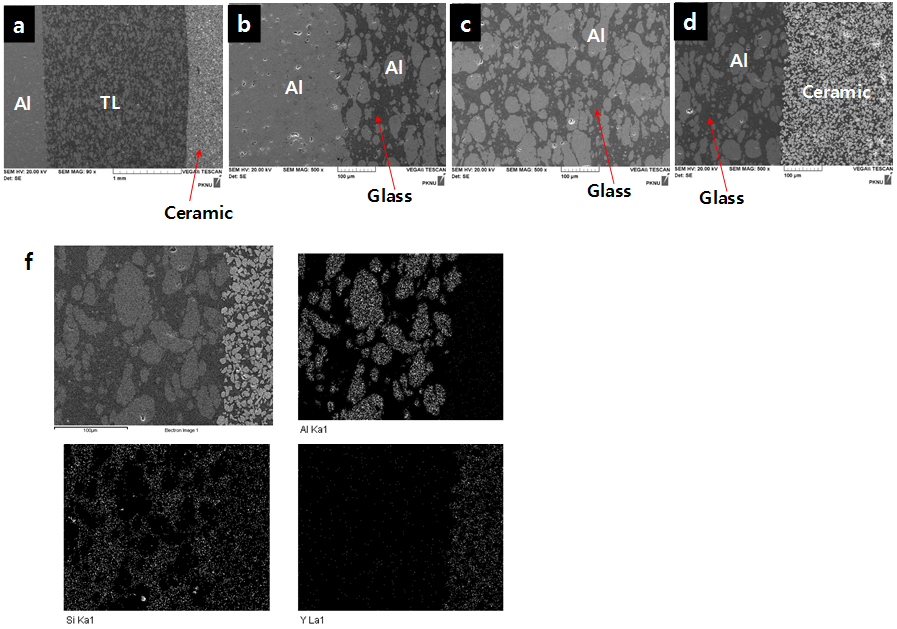


**Figure S4.** SEM images of ACC2. (a) Al, TL, and ceramic regions of ACC2. The TL (2 mm thickness) comprises 50 vol% Al and 50 vol% glass. The ceramic layer comprises 50 vol% YAG:Ce and 50 vol% glass. (b) Al and TL regions, (c) TL region, (d) TL and ceramic regions of ACC2. (f) Element mapping images of ACC2.


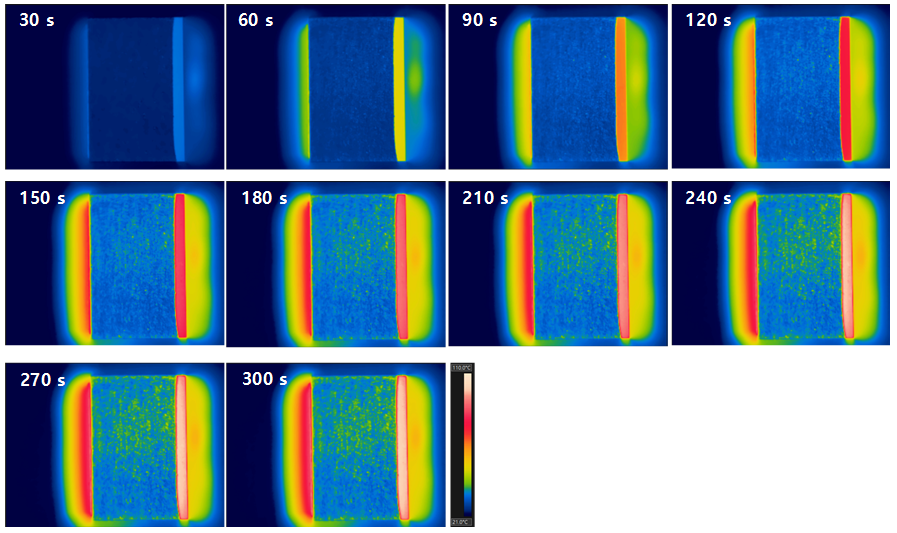


**Figure S5.** Images of the temperature distribution of ACC1 during the operation of a 4 W-blue LD over time.


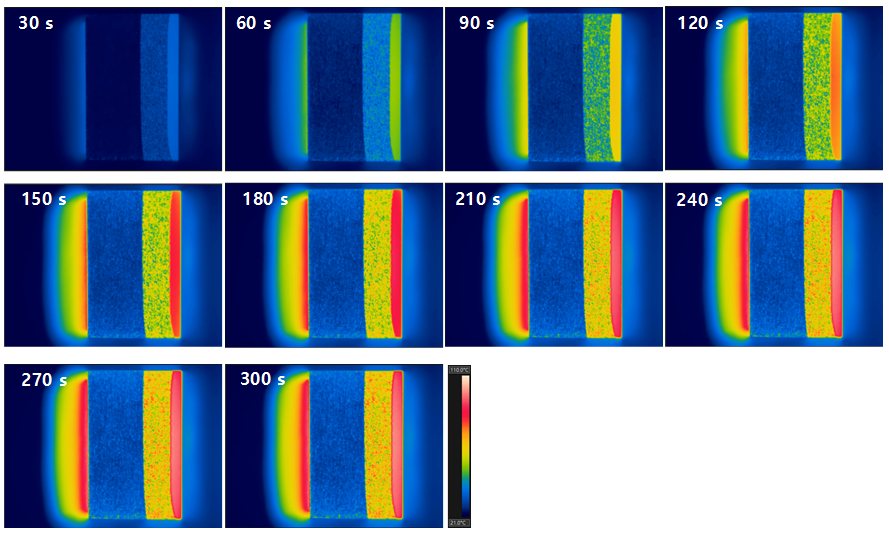


**Figure S6.** Images of the temperature distribution of ACC2 during the operation of 4 W-blue LD over time.


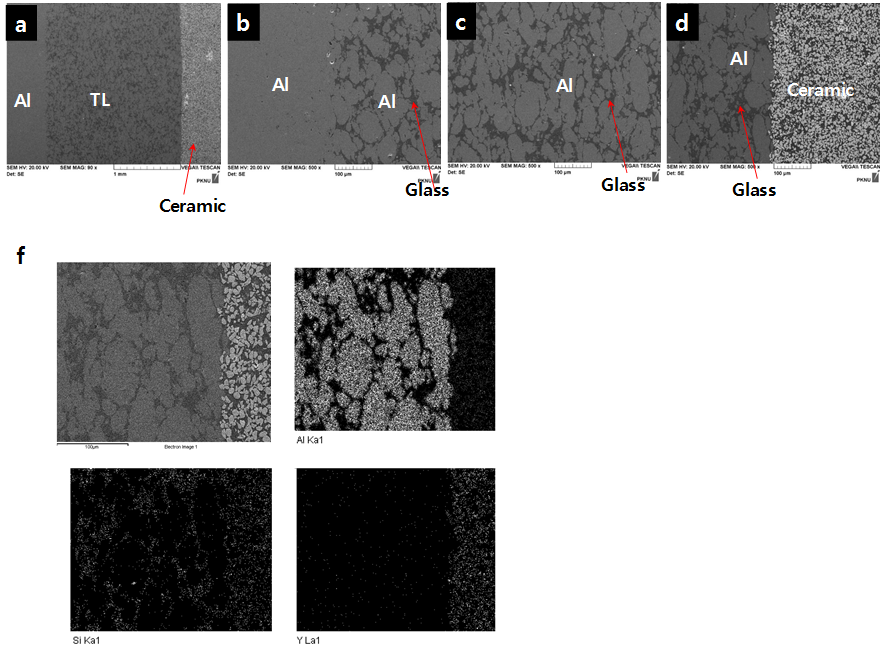


**Figure S7.** SEM images of ACC3. (a) Al, TL, and ceramic regions of ACC3. The TL (2 mm thickness) comprises 75 vol% Al and 25 vol% glass. The ceramic layer comprises 50 vol% YAG:Ce and 50 vol% glass. (b) Al and TL regions, (c) TL region, (d) TL and ceramic regions of ACC3. (f) Element mapping images of ACC3.


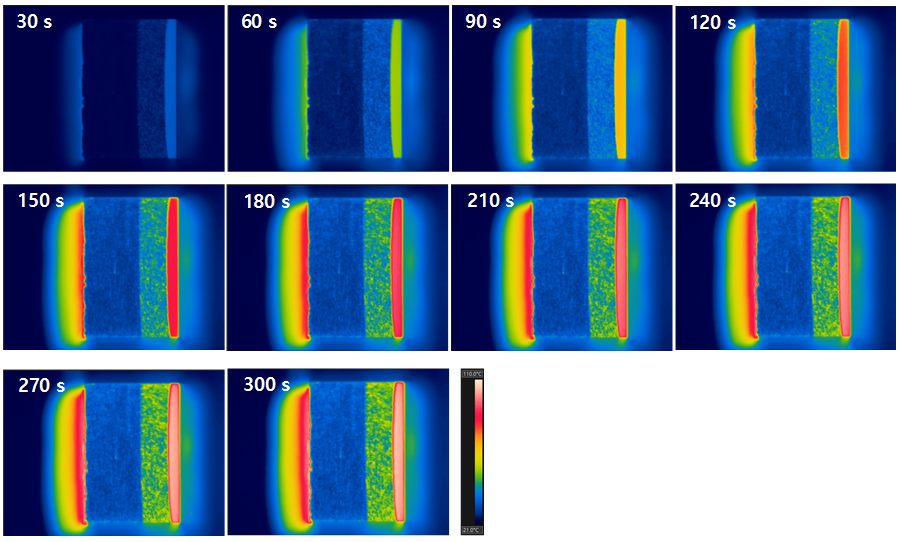


**Figure S8.** Images of the temperature distribution of ACC3 during the operation of 4 W-blue LD over time.


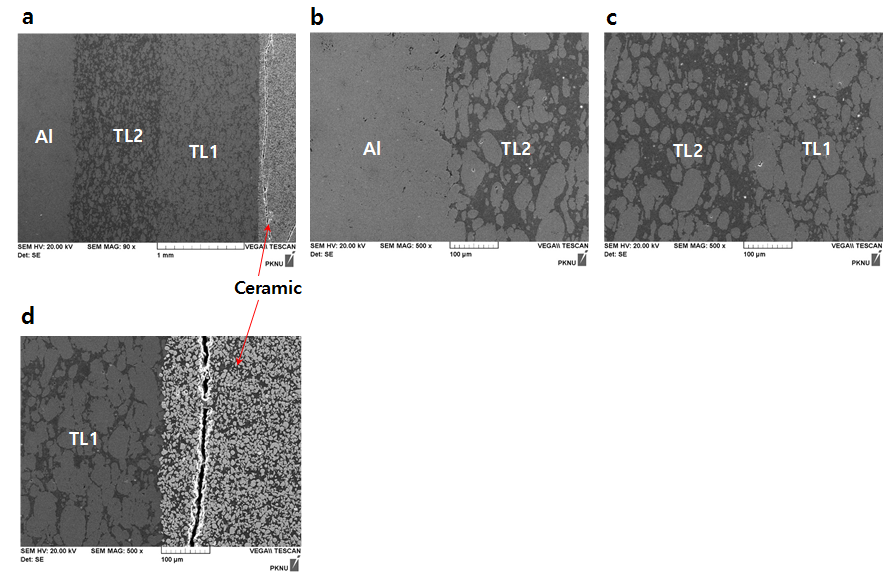


**Figure S9.** SEM images of ACC4. (a) Al, TL1, TL2, and ceramic regions of ACC4. TL1 (approximately 1 mm thickness) comprises 75 vol% Al and 25 vol% glass. TL2 (approximately 1 mm thickness) comprises 50 vol% Al and 50 vol% glass. The ceramic layer comprises 50 vol% YAG:Ce and 50 vol% glass. (b) Al and TL2 regions, (c) TL2 and TL1 regions, (d) TL1 and ceramic regions of ACC4.


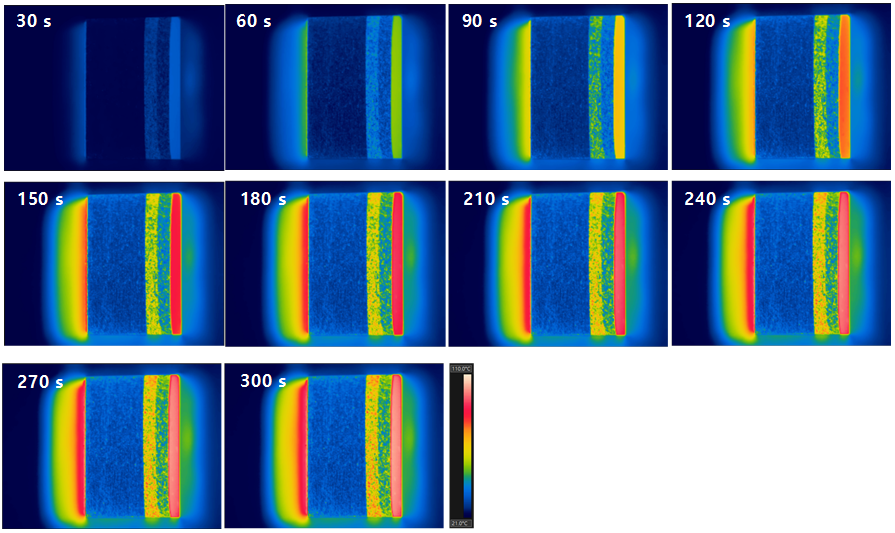


**Figure S10.** Images of the temperature distribution of ACC4 during the operation of a 4 W-blue LD over time.
